# Data of the release properties of astaxanthin-loaded zein/calcium alginate composite microparticles in fatty food simulant system at 4 °C and 25 °C

**DOI:** 10.1016/j.dib.2020.106392

**Published:** 2020-10-09

**Authors:** Ru Song, Yu Qi, Zhe Jia, Xinyan Liu, Rongbian Wei

**Affiliations:** a School of Food Science and Pharmacy, Zhejiang Ocean University, Zhoushan 316022, China; b School of Fishery, Zhejiang Ocean University, Zhoushan 316022, China; c School of Marine Science and Technology, Zhejiang Ocean University, Zhoushan 316022, China

**Keywords:** Shrimp by-products, Astaxanthin, Zein/calcium alginate, Composite microparticles, Fatty food simulant system, Cumulative release, Release kinetics

## Abstract

Recently, we demonstrated the characteristics and molecular interactions of Astaxanthin (Asta), extracted from shrimp (*Litopenaeus vannamei*) by-products to zein/calcium alginate (CA) (named as Asta-loaded zein/CA composite microparticles). The encapsulation efficiency of Asta-zein/CA composite microparticles obtained from freeze dried, 40 °C or 50 °C oven dried was across 80% [1]. In this data, we investigted the release properties of Asta-loaded zein/CA composite microparticles in simulating fatty food system (95% ethanol solution) at 4  °C and 25  °C. At different points of time, the cumulative release percentages of Asta from the tested composite microparticles were calculated. The release kinetics of Asta from the composite microparticles was investigated using Zero order, First order, Higuchi and Rigter-Peppas models. We observed all of the tested composited microparticles displayed an initial burst effect followed by subsequent attenuating release in 4 °C and 25 °C fatty food simulant system. At 4 °C fatty food system, the Asta released from 40 °C oven dried and 50 °C oven dried composite microparticles fit best with First-order and Ritger-Peppas models, respectively. At 25 °C fatty food system, all of these tested composite microparticles fit best with Higuchi model. Our results indicate the prepared composite microparticles are expected to be used as a delivery carrier for restrained release of antioxidant Asta in fatty foods, such as in natural vegetable oils or fried foods.

## Specifications Table

**Table:** 

Subject	Food Science
Specific subject area	Food microparticles and release property
Type of data	Table Figure
How data were acquired	Under a fatty food simulant system (95% ethanol solution), real time measuring of the Asta cumulative release from the tested composite microparticles were performed at 4 °C and 25 °C fatty food simulant system.
Data format	Raw Analyzed
Parameters for data collection	Asta-loaded zein/CA composite microparticles (freeze dried, 40 °C oven dried and 50 °C oven dried) were added into the fatty food simulant system and stored at 4 °C and 25 °C in the darkness. At time intervals (0–23 h), the cumulative release percentage of Asta was measured.
Description of data collection	At different points of time, the released Asta content was determined by measuring the absorbance at 477 nm [1], and then the Asta cumulative release percentages of all tested composite microparticles were calculated. All measurements were performed in triplicates. Based on the results of Asta cumulative release percentages, the release kinetics were obtained by fitting Zero order, First order, Higuchi and Rigter-Peppas models associated with diffusion.
Data source location	School of Food Science and Pharmacy, Zhejiang Ocean University, Zhoushan, China.
Data accessibility	All relevant data is included in the article.
Related research article	R. Song, Y. Qi, Z. Jia, X. Liu, R. Wei, Astaxanthin-loaded zein/calcium alginate composite m icroparticles: characterization, molecular interaction and release kinetics in fatty food simulant system, LWT-Food Sci. Technol. In Press.

## Value of the Data

• These data provide useful insights into the release properties of Asta-loaded zein/CA composite microparticles in fatty food simulant system.
• Food scientist and nutritionist can benefit from these data.
• These data can provide useful information on diffusion mechanism of Asta-loaded zein/CA composite microparticles in fatty food system when stored at 4 °C and room temperatures.
• These data will provide enhanced understanding of potential applications of Asta-loaded zein/CA composite microparticles in natural vegetable oils or fried foods.

## 1 Data Description

Detail raw data of the cumulative release percentages of Asta from the Asta-loaded zein/CA composite microparticles at different time points in fatty food simulant system at 4 °C and 25 °C were summarized in Table 1 and Table 2. Data obtained from Table 1 to Table 2 were evaluated for the average cumulative release percentages of Asta from all tested composite microparticels when incubated in fatty food simulant system at 4 °C (Table 3) and 25 °C (Table 4), respectively.

**Table 1:** Detail data of the cumulative release percentages of Asta from the Asta-loaded zein/CA composite microparticles incubation of different time in fatty food simulant system at 4 °C.

Time/h	Free dried	40 °**C** oven dried	50 °**C** oven dried
0	7.16	0.00	0.00
0	16.57	0.00	0.00
0	18.46	0.00	0.00
0.33 (20 min)	97.08	27.18	15.43
0.33 (20 min)	95.19	33.55	31.38
0.33 (20 min)	100.84	22.93	20.74
0.67 (40 min)	82.22	42.70	20.33
0.67 (40 min)	110.47	51.20	36.29
0.67 (40 min)	102.94	38.46	17.68
1.00 (60 min)	77.79	44.71	17.27
1.00 (60 min)	83.44	42.59	33.23
1.00 (60 min)	96.62	38.77	30.57
1.33 (80 min)		50.33	35.48
1.33 (80 min)		60.95	59.41
1.33 (80 min)		56.70	35.48
1.67 (100 min)		42.15	33.83
1.67 (100 min)		63.38	33.83
1.67 (100 min)		40.03	31.17
2		56.12	30.76
2		62.49	44.06
2		60.37	38.74
3		51.76	35.94
3		68.75	43.92
3		62.38	49.24
5		78.97	52.02
5		98.07	(91.92)
5		89.58	65.32
7		87.34	54.54
7		100.08	(91.77)
7		87.34	67.84
11		92.16	64.68
11		93.25	(110.02)
11		91.16	80.64
17		87.69	88.48
17		102.55	83.16
17		104.67	83.16
23		101.65	(40.35)
23		93.16	88.33
23		101.65	83.01

Note: Time in brackets were actural incubation time. Raw data in brackets were deleted in calculating the mean value and standard deviation due to high deviation.

**Table 2:** Detail data of the cumulative release percentages of Asta from the Asta-loaded zein/CA composite microparticles incubation of different time in fatty food simulant system at 25 °C.

Time/h	Free dried	40 °**C** oven dried	50 °C oven dried
0	16.57	0.00	0.00
0	12.81	0.00	0.00
0	9.04	0.00	0.00
0.33 (20 min)	72.47	25.05	10.11
0.33 (20 min)	(25.39)	22.93	4.79
0.33 (20 min)	76.23	39.91	4.79
0.67 (40 min)	119.57	47.09	21.40
0.67 (40 min)	106.39	51.34	16.08
0.67 (40 min)	87.56	34.35	32.04
1.00 (60 min)	(60.36)	51.22	50.25
1.00 (60 min)	95.43	61.84	31.63
1.00 (60 min)	116.15	46.98	50.25
1.33 (80 min)		62.22	43.72
1.33 (80 min)		55.85	(10.42)
1.33 (80 min)		62.22	59.68
1.67 (100 min)		54.32	42.16
1.67 (100 min)		43.71	42.16
1.67 (100 min)		75.55	63.44
2		60.58	46.01
2		75.44	(23.39)
2		43.59	46.01
3		70.87	45.87
3		79.36	53.84
3		64.50	67.14
5		60.71	47.06
5		81.94	70.99
5		69.20	70.99
7		94.56	101.79
7		115.79	96.47
7		98.81	96.47

Note: Time in brackets were actural incubation time. Raw data in brackets were deleted in calculating the mean value and standard deviation due to high deviation.

**Table 3:** Estimation of the average cumulative release percentages of Asta at different time incubation in 4 °C fatty food simulant system.

Time/h	Free dried	40 °C oven dried	50 °C oven dried
0	14.06	0.00	0.00
0.33 (20 min)	97.70	27.88	22.52
0.67 (40 min)	98.55	44.12	24.77
1.00 (60 min)	85.95	42.02	27.02
1.33 (80 min)		55.99	43.46
1.67 (100 min)		48.52	32.94
2		59.66	37.85
3		60.96	43.03
5		88.87	58.67
7		91.59	61.19
11		92.19	72.66
17		98.30	84.93
23		98.82	85.67

**Table 4:** Estimation of the average cumulative release percentages of Asta at different time incubation in 25 °C fatty food simulant system.

Time/h	Free dried	40 °C oven dried	50 °C oven dried
0	12.81	0.00	0.00
0.33 (20 min)	74.35	29.30	6.56
0.67 (40 min)	104.51	44.26	23.17
1.00 (60 min)	105.79	53.35	44.04
1.30 (80 min)		60.10	51.70
1.67 (100 min)		57.86	49.26
2		59.87	46.01
3		71.58	55.62
5		70.61	63.01
7		103.05	98.24

Table 3 shows an initial burst effect followed by subsequent slower release for all of these composite microparticles. For freeze dried composite microparticles, 97.70% of Asta was released during 20 min of burst effect stage. The cumulative release of Asta for the 40 °C oven dried composite microparticles was 88.87% at the end of its burst effect at 5 h. The 50 °C oven dried composite microparticles showed lower Asta cumulative release percentage than the other composite microparticles in the 4 °C fatty food simulant system. Table 4 shows that the freeze dried composite microparticles showed higher Asta release percentage than the 40 °C oven dried and 50 °C oven dried ones in 25 °C fatty food simulant system under same incubation time. A lower Asta release percentage was found in the 50 °C ven dried composite microparticles.

Based on the data of Table 3 and Table 4, the release kinetics of Asta from all tested composite microparticles were measured to fit Zero order, First order, Higuchi and Rigter-Peppas models.

**Fig. 1:**
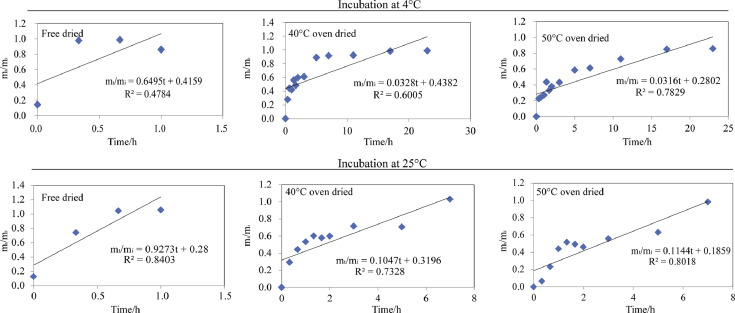
Zero order model fitting for all the tested composite microparticles.

Fig. 1. describes the Zero order model (m_t_/m_i_=at+*b*) prediction for the Asta cumulative release from freeze dried, 40 °C oven dried and 50 °C oven dried composite microparticles when incubated in fatty food simulant system under 4 °C and 25 °C. The compsotie microparticles showed higher release kinetics parameters (*R*^2^) in 25 °C fatty food simulant system than the corresponding ones in 4 °C fatty food simulant system.

**Fig. 2:**
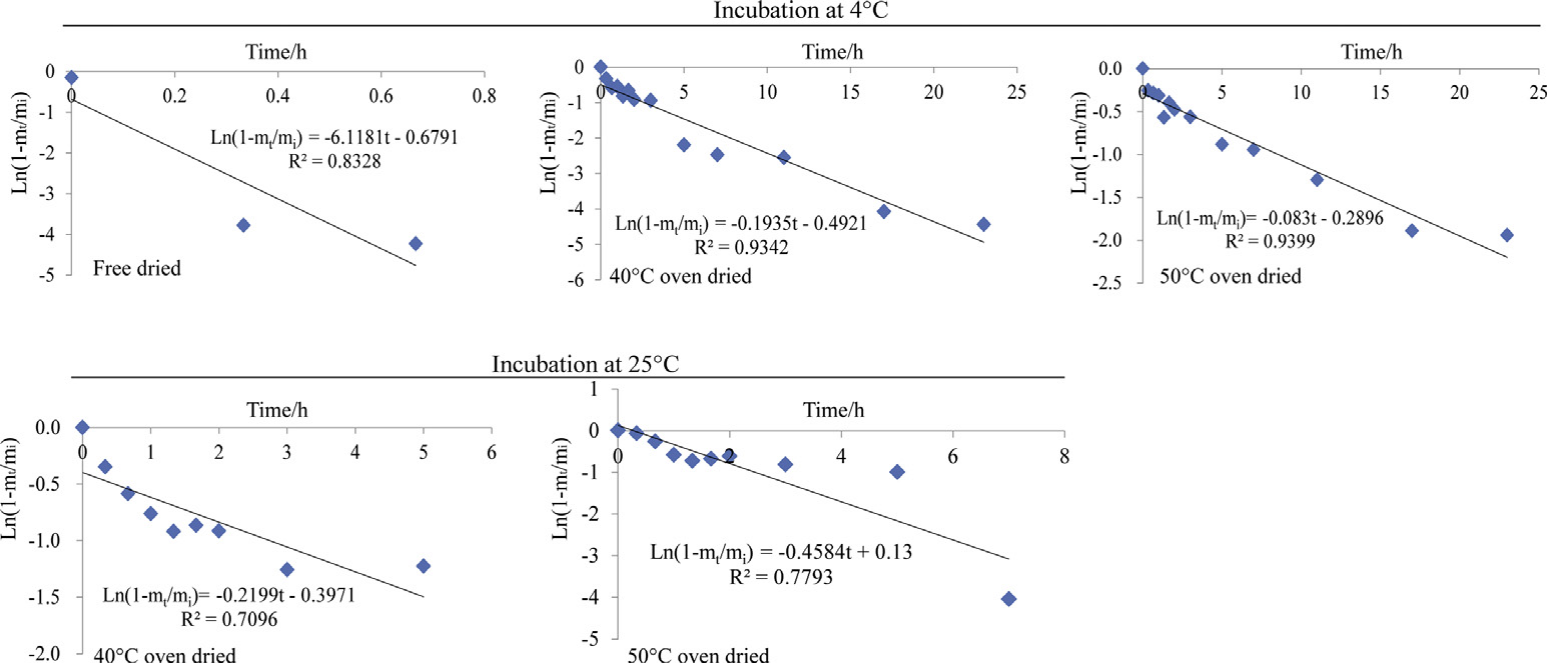
First-order model fitting for all the tested composite microparticles.

Fig. 2. describes the First order model (Ln(1-m_t_/m_i_)=at+*b*) fitting for the Asta cumulative release from freeze dried, 40 °C oven dried and 50 °C oven dried composite microparticles when incubated in fatty food simulant system under 4 °C and 25 °C. The 40 °C oven dried and 50 °C oven dried composite microparticles in 4 °C fatty food simulant system showed higher release kinetic parameters (*R*^2^ > 0.93) than other microparticles. In 25 °C fatty food simulant system, no enough data were obtained in the free dried composite, due to the average cumulative release percentages across 100% at 0.67 h and 1.0 h of incubation.

**Fig. 3:**
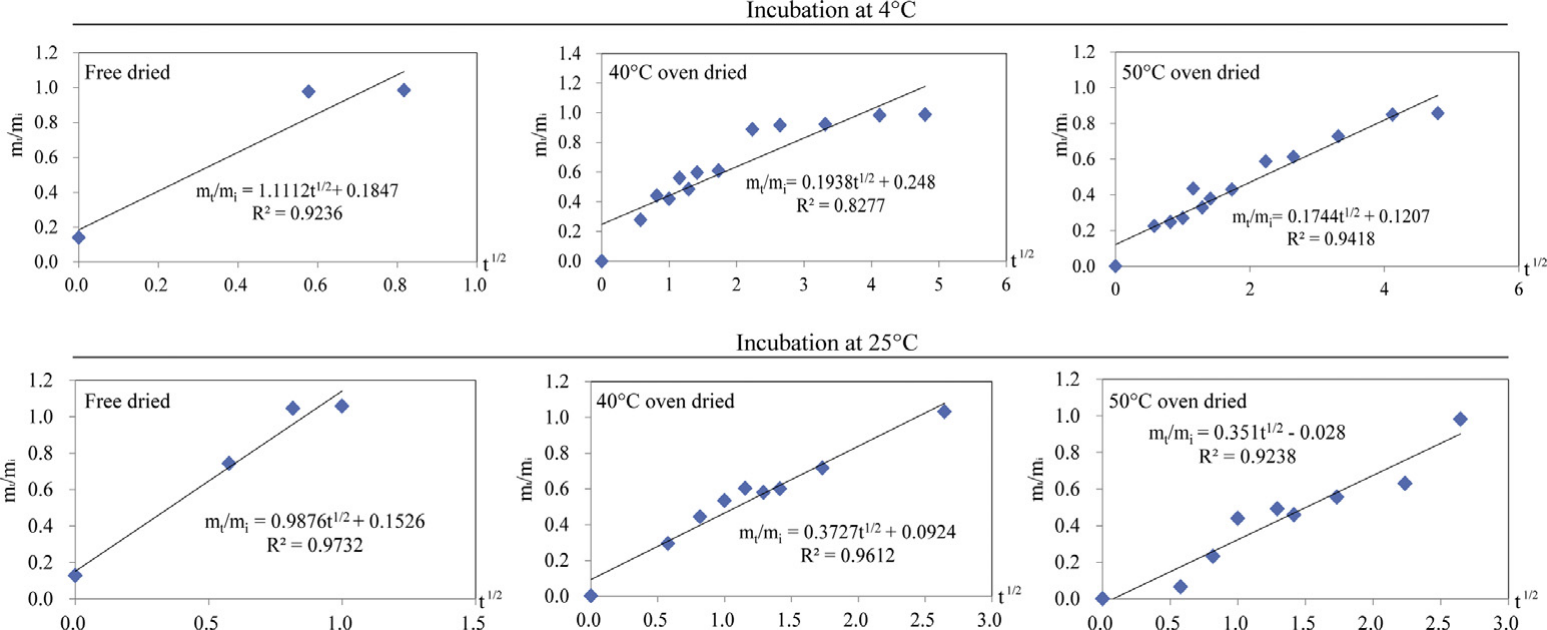
Higuchi model fitting for all the tested composite microparticles.

Fig. 3. shows the Higuchi model (m_t_/m_i_=at^1/2^ + *b*) fitting for the Asta cumulative release from freeze dried, 40 °C oven dried and 50 °C oven dried composite microparticles when incubated in 4 °C or 25 °C fatty food simulant system. Except for the 40 °C oven dried composite microparticles incubated at 4 °C fatty food simulant system, the Asta release kinetics parameters (*R*^2^) were across 0.92 for the other microparticles in Higuchi model.

**Fig. 4:**
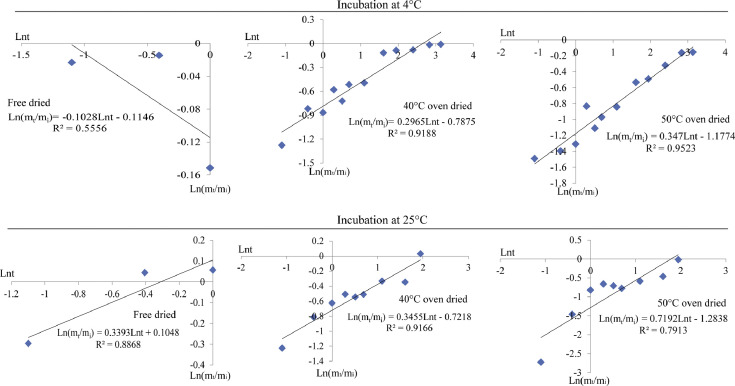
Rigter-Peppas model fitting for all the tested composite microparticles.

Fig. 4. describes the Rigter-Peppas model (Ln(m_t_/m_i_)=aLnt+*b*) fitting for the Asta cumulative release from freeze dried, 40 °C oven dried and 50 °C oven dried composite microparticles in fatty food simulant system. In 4 °C fatty food simulant system, the release kinetics parameters (*R*^2^) were over 0.90 for the 40 °C oven dried and 50 °C oven dried composite microparticles. In 25 °C fatty food simulant system, the Asta cumulative release of 40 °C oven dried composite microparticles fit better with the Rigter-Peppas model (*R*^2^=0.9166).

## 2 Experimental Design, Materials and Methods

### 2.1 Materials and methods

Free Asta standard (≥ 98%, HPLC) was purchased from Aladdin Industrial Corporation (Shanghai, China). Asta-loaded zein/CA astaxanthin composite microparticles used in this data article (freeze dried, 40 °C oven dried and 50 °C oven dried) were prepared described in our co-submitted related research article [1].

### 2.2 Asta content determination

The Asta extracts have specific absorbance at 477 nm [2]. An Asta standard calibration curve (*y* = 0.0946x+0.0108, *R*^2^=0.9926) was determined by measuring the absorbance (y) of Asta ethanol solutions (x) (concentration from 0 to 10 μg/mL) at 477 nm using a 1510micro-plate reader (Thermo Fisher Scientific Oy, Vantaa, Finland). The Asta content (μg) of tested solution was quantified by multiply the Asta concentration (μg/mL), determined by measuring absorbance at 477 nm, with the solution volume (mL).

### 2.3 Release property in fatty food simulant system

The oxidative products of natural vegetable oil formed during storage have specific absorbance at 390 nm to 550 nm, which could affect the Asta quantification by spectrophotometry at 477 nm. Therefore, the 95% ethanol solution (v/v) was used to stimulate the fatty food system [3]. Approximately 50 mg of the composite microparticles were blended with 10 mL of 95% ethanol solution in a 25 mL weighing bottle, and kept at 4 °C and 25 °C in darkness. At different time intervals, i.e. 0 to 23 h for 4 °C treated groups, and 0 to 7 h for 25 °C treated groups, 1 mL of solution was pipetted out and replaced with 1 mL of fresh 95% ethanol solution to remain constant volume. After centrifuged at 5000 *×* *g* for 10 min, the resulted supernatants were collected and measured the absorbance of 477 nm, followed by calculation of the cumulative content of Asta released in 10 mL of fatty food system. The cumulative release percentage of Asta at different time points was determined as follows:

$$\text{Cumulative}\;\text{release}/\%=(m_{t}/m_{i})\times 100$$

*m_i_*: the initial Asta content (μg) in the composite microparticles; *m_t_*: the cumulative Asta content (μg) at each time point. All measurements were performed in triplicates. Data were expressed as mean ± standard deviation.

### 2.4 Release kinetics of Asta from the composite microparticles

After determining the cumulative release percentage of Asta at different time points, the release kinetics of Asta from the composite microparticles were investigated by fitting four models, i.e. Zero order, First order, Higuchi and Rigter-Peppas.

$$\text{Zero}\;\text{order}:\;m_{t}/m_{i}=at+b;$$

$$\text{First}\;\text{order}:\;\text{Ln}\left(1-m_{t}/m_{i}\right)=at+b;$$

$$\text{Higuchi}:\;m_{t}/m_{i}=at^{1/2}+b;$$

$$\text{Rigter}-\text{Peppas}:\;\text{Ln}(m_{t}/m_{i})=aLnt+b.$$

## Declaration of Competing Interest

The authors declare that they have no known competing financial interests or personal relationships which have, or could be perceived to have, influenced the work reported in this article.
